# Revealing Invisible Water: Moisture Recycling as an Ecosystem Service

**DOI:** 10.1371/journal.pone.0151993

**Published:** 2016-03-21

**Authors:** Patrick W. Keys, Lan Wang-Erlandsson, Line J. Gordon

**Affiliations:** 1 Stockholm Resilience Centre, Stockholm University, Stockholm, Sweden; 2 Department of Atmospheric Science, Colorado State University, Fort Collins, United States of America; 3 Department of Water Management, Faculty of Civil Engineering and Geosciences, Delft University of Technology, Delft, The Netherlands; University of Brighton, UNITED KINGDOM

## Abstract

An ecosystem service is a benefit derived by humanity that can be traced back to an ecological process. Although ecosystem services related to surface water have been thoroughly described, the relationship between atmospheric water and ecosystem services has been mostly neglected, and perhaps misunderstood. Recent advances in land-atmosphere modeling have revealed the importance of terrestrial ecosystems for moisture recycling. In this paper, we analyze the extent to which vegetation sustains the supply of atmospheric moisture and precipitation for downwind beneficiaries, globally. We simulate land-surface evaporation with a global hydrology model and track changes to moisture recycling using an atmospheric moisture budget model, and we define vegetation-regulated moisture recycling as the difference in moisture recycling between current vegetation and a hypothetical desert world. Our results show that nearly a fifth of annual average precipitation falling on land is from vegetation-regulated moisture recycling, but the global variability is large, with many places receiving nearly half their precipitation from this ecosystem service. The largest potential impacts for changes to this ecosystem service are land-use changes across temperate regions in North America and Russia. Likewise, in semi-arid regions reliant on rainfed agricultural production, land-use change that even modestly reduces evaporation and subsequent precipitation, could significantly affect human well-being. We also present a regional case study in the Mato Grosso region of Brazil, where we identify the specific moisture recycling ecosystem services associated with the vegetation in Mato Grosso. We find that Mato Grosso vegetation regulates some internal precipitation, with a diffuse region of benefit downwind, primarily to the south and east, including the La Plata River basin and the megacities of Sao Paulo and Rio de Janeiro. We synthesize our global and regional results into a generalized framework for describing moisture recycling as an ecosystem service. We conclude that future work ought to disentangle whether and how this vegetation-regulated moisture recycling interacts with other ecosystem services, so that trade-offs can be assessed in a comprehensive and sustainable manner.

## Introduction

An ecosystem service is a benefit derived by society that can be traced back to an ecological process [1], and water is often an important part of ecosystem service assessments [2]. Surface waters such as lakes, rivers and wetlands are regularly included in ecosystem service inventories, as providing fish habitat, flood mitigation, and maintaining water quality [3, 4]. However, evaporation from ecosystems, and the subsequent water vapor that flows outward from those ecosystems, has been largely ignored by the ecosystem services community. In most instances where evaporation has been considered, it has been perceived as neutral or detrimental to the generation of ecosystem services, particularly with regard to agricultural production where evaporation is often referred to as a net water loss [5].

However, water that leaves the earth’s surface as evaporation does not disappear, but rather flows through the atmosphere as water vapor, and eventually falls out as precipitation, such as rain or snow [6]. In other words, moisture is recycled from the point of evaporation, through the atmosphere, to downwind locations where it becomes precipitation. Global model simulations have revealed that on average 40% of global annual rainfall comes from upwind, land evaporation, but this number varies significantly, and can be much higher in certain places, e.g. China [7]. Many places that receive moisture from ecosystems upwind are economically dependent on rainfed activities, implying the importance of the regulation of moisture by upwind ecosystems to downwind societies [8, 9]. Importantly, land-use change is altering many of those upwind ecosystems, with significant effects on evaporation [10, 11].

These changes in evaporation in one location can significantly alter the precipitation that eventually falls downwind [12–14]. Furthermore, there is very low spatial variability between the upwind sources and downwind sinks of moisture recycling [15]. As a result, there are important trade-offs that might emerge when considering land-use decisions in one location, and how those decisions impact people and places in another location. In order to handle environmental decisions that require evaluating tradeoffs between multiple options of land-use change, a growing community of scientists and policy-makers employ ecosystem service frameworks [16, 17]. However, since vegetation-regulated moisture recycling has not been integrated into ecosystem service frameworks, it is not included in these evaluations of land-use change trade-offs.

Here, we argue that Vegetation-regulated Moisture Recycling (VMR) is a critical ecosystem service that must be quantified and evaluated for its relative importance around the planet. We define VMR as the evaporated water that returns as precipitation downwind that is attributable to vegetation on land. VMR can thus be estimated as the amount of water that is regulated by current (i.e. present-day) vegetation relative to desert vegetation, in terms of both (a) the vegetation-regulated evaporation that enters the atmosphere, and (b) the fraction of precipitation falling downwind that can be attributed to upwind vegetation.

We present a method for quantifying where VMR provides a significant ecosystem service. We use this method in an idealized comparison of a global simulation of current vegetation with a global simulation where all terrestrial surfaces are converted to desert vegetation. These global simulations involve both a land-surface hydrology model that simulates evaporation [18], and an atmospheric moisture budget model that tracks where moisture enters the atmosphere as evaporation, where it flows around the planet, and where it eventually falls out as precipitation [19]. Our results depict the importance of VMR in terms of the generation of the ecosystem service (i.e. VMR sources), and in terms of the potential beneficiaries of the ecosystem service (i.e. VMR sinks). Furthermore, we explore how our method could be used in a more practical case study, and synthesize our findings in a generalized framework.

The novelty of our work is not in the moisture recycling analysis, nor in the idealized global simulation of current vegetation versus desert vegetation. Rather, the novelty is (a) our integration of moisture recycling and VMR into a quantified ecosystem services approach, and (b) the generalization of these findings into a framework that can be broadly applied using different modeling setups or different sources of data.

## Materials and Methods

We use a coupled land surface and atmospheric moisture budget model in this research. We employ the Simple Terrestrial Evaporation to Atmosphere Model (hereafter, STEAM) which is a land-surface hydrology model that is specifically designed to realistically partition evaporation fluxes to the atmosphere. STEAM has been described in detail in previous work so we provide only a brief description below, and direct the reader towards the full model documentation [18]. The Water Accounting Model 2layers (hereafter, WAM-2layers) is an atmospheric moisture budget model that tracks water from its evaporative origin on the planet, through the atmosphere, and to its fate as downwind precipitation. The Water Accounting Model (both 1 and 2 layer versions) has been thoroughly described and applied in previous work [7, 8, 15, 20–23], so we provide a summary description below. Finally, the coupling procedure that we employ in our research has been previously explained, so we provide only a brief overview below [19].

### STEAM

STEAM uses several meteorological and soil variables as inputs to estimate evaporation from five different stocks of accumulated water on the land surface. The driving datasets for STEAM include meteorological data from the ERA-Interim data archive, including evaporation, precipitation, snowfall, snowmelt, temperature, dew-point, wind speed, and short- and long- wave radiation [24]. The data span the time period 1997 to 2014, and were downloaded at the 3 hourly timestep, at a resolution of 1.5° × 1.5° grid resolution. The land surface data come from the Moderate Resolution Imaging Spetroradiometer dataset (MODIS), and are based on the International Geosphere Biosphere Programme land-use classes [25]. The soil data is based on the Harmonized World Soil Database. The evaporation partitioning that STEAM performs is divided into five key steps. First, the vegetation canopy intercepts rainfall, and accumulates and evaporates water. Second, as water drips off the canopy, it is intercepted by the soil surface and evaporates. Third, as water saturates the soil surface, it can be partitioned as moisture in the soil column or taken up by plant roots and transpired. Fourth, excess water can pool as open water bodies and evaporate. Fifth, water can freeze and accumulate until it is melted, and eventually evaporates.

The evaporation values that are generated by STEAM have been compared against several existing products, in whole and in part. For example, the total evaporation values (sum of all partitioned fluxes) are within the interquartile range of the global, multi-model synthesis product LandFlux-EVAL. A rigorous and detailed evaluation of STEAM, relative to other evaporation estimates, found that it performs well in estimating total evaporation, individual partitioned fluxes (e.g. vegetation interception), and across different vegetation types [18].

### WAM-2layers

Given that we want to know where land evaporation later falls as precipitation on land downwind, we must be able to track moisture around the planet. There are many moisture budget or tracking approaches [26–28], and we employ the Water Accounting Model, 2layers (hereafter, WAM-2layers) to perform our global moisture tracking analysis [19]. The WAM-2layers tracks the flow of moisture around the planet using an Eulerian approach. Imagine a column of air above a given location. At each time step, the WAM-2layers keeps track of how much water enters the atmosphere as evaporation, as well as how much water exits the atmosphere as precipitation. In between each time-step, the WAM-2layers calculates how much water moves between each grid cell in each of the four cardinal directions. Thus, as we step forward in time the WAM-2layers provides an accounting of how much moisture enters and exits the atmosphere, as well as where it travels horizontally in the atmosphere, for all locations globally. As the name suggests, the WAM-2layers has two atmospheric layers, meaning there is a layer of the atmosphere close to the surface of the earth, as well a layer higher in the atmosphere. The purpose of this is to capture the varying wind speeds at different altitudes in the atmosphere, i.e. wind shear. The WAM-2layers requires six variables as input for its calculation. In this paper, we use the ERA-Interim data, which was downloaded at the 1.5° × 1.5° grid resolution [24]. These data include: 6-hourly winds (zonal and meridional) and relative humidity; 6-hourly surface pressure; and 3-hourly precipitation and evaporation. The model is run at the 15-minute time step to eliminate numerical errors, and thus the data are discretized to the 15-minute time step, using linear interpolation. The data span the period January 1997 to December 2014. The Water Accounting Model (one and two layer versions) has been used extensively [7, 8, 20–23], including in a favorable comparison with an RCM [21]. Likewise, WAM-2layers has also been used with other driving data (the Modern Era Retrospective Analysis for Research, MERRA) to explore its sensitivity to multiple data sources, as well as to understand moisture recycling variability [15].

### Model coupling

Coupling STEAM and WAM-2layers requires two key steps. First, STEAM’s simulated evaporation is substituted for the ERA-Interim evaporation in WAM-2layers. Second the changes in moisture input (i.e. evaporation) are propagated and tracked using WAM-2layers, resulting in modified precipitation [19]. Also, given that land-use change will change evaporation, and subsequently the moisture available for precipitation, we must run the STEAM and WAM-2layers for several iterations so that the new moisture budget converges. We define convergence as achieving a less than 1% difference in annual precipitation for every grid-cell between consecutive model runs. Hereafter, we refer to the coupling between STEAM and WAM-2layers as STEAM+WAM. Using STEAM+WAM we run two land-use scenarios: current vegetation (based on MODIS land-cover) and desert vegetation (by changing all land-surfaces globally to the ‘barren’ land class in STEAM, which includes sparse, desert vegetation). In the desert vegetation scenario, all land parameters are changed (e.g. minimum and maximum leaf area index, root depth, albedo, etc.). As a result, all aspects of the evaporation partitioning regime will be changed, including all five partitions (canopy interception, ground interception, soil moisture evaporation, transpiration, and open water evaporation). We run both scenarios for 18 years, using the first three years as “model spin-up”, leaving 15 years for analysis (2000–2014). In this way, we are able to isolate the impact of changes to evaporation on global moisture recycling patterns.

### Note on the scope of coupling procedure

In this paper, we only study the land-use change effects on rainfall through moisture recycling.

However, there are also other more complex interactions between land and atmosphere at play, e.g. land-use change effects on the atmosphere’s thermal structure, changes to atmospheric circulation, and interactions with monsoon systems. While reductions in precipitation always follow from reductions in evaporation in moisture recycling, these reductions in precipitation can also follow from increases in evaporation that then drive other types of land-atmosphere interactions (e.g. irrigation changing land-ocean temperature gradients, and altering monsoon onset [29, 30]). These interactions act all at once, but the dominating mechanism depends on the spatial scale of change as well as on the region of change. Studies have shown that local scale (100–1000 km) perturbations are important for the thermal structure, while moisture recycling operates at the regional scale (larger than 1000 km), and atmospheric circulation is modified by changes at the regional to global scale [20, 31, 32]. Obviously, the desert vegetation scenario we simulated is not, nor aims to be, realistic. Rather, the purpose of it is to provide a theoretical baseline for the vegetation-regulated moisture recycling calculation. Finally, the effects of land use change on precipitation are difficult to simulate with fully coupled climate models, due to noise and model uncertainty [33]. Although simplified, the STEAM+WAM coupled simulation isolates the role of changing land-use on changes to evaporation, with subsequent changes to precipitation. Since the circulation, ocean evaporation, and climate aspects are kept identical in the scenarios, we can attribute any changes we see in atmospheric moisture content, or eventual downwind precipitation, to changes in evaporation.

### Calculation of VMR ecosystem services

A key contribution of this work is the method we introduce to quantify vegetation-regulated moisture recycling (VMR) ecosystem services. Given that the service is generated in one place (i.e. sources of VMR) and is potentially realized as a benefit in another place (i.e. sinks of VMR), we provide a detailed overview of our calculation methods.

#### Sources of VMR

First, we identify the evaporation recycling ratio by identifying the fraction of evaporation from a given grid-cell that returns as rainfall downwind; note that we are using the same approach used in previous studies [7, 8]. The evaporation recycling ratio is defined for each grid cell as,

$$\begin{array}{c} \frac{E_{\text{c,track}}}{E_{\text{c}}}=\varepsilon_{\text{c}} \end{array}$$ (1)

where, *E*_c,track_ is the evaporation from current vegetation that returns to land as precipitation downwind, *E*_c_ is the total evaporation from current vegetation, and *ε*_c_ is the terrestrial evaporation recycling ratio for current vegetation.

Second, we are able to identify the extent to which vegetation regulates evaporation flows, by subtracting the tracked evaporation from the desert vegetation scenario from the tracked evaporation from the current vegetation scenario, and dividing this by the total evaporation, from current vegetation. This generates a ratio we call vegetation regulation, where for each grid cell,

$$\begin{array}{c} \frac{E_{\text{current}}-E_{\text{desert}}}{E_{\text{current}}}=V_{E} \end{array}$$ (2)

where, *E*_current_ is the amount of evaporation that is recycled downwind under current vegetation, *E*_desert_ is the amount of evaporation that is recycled downwind under desert vegetation, and *V*_*E*_ is the fraction of evaporation that is vegetation-regulated.

To better understand what this ecosystem service means in a tangible sense we can combine the previous information to generate a global distribution of vegetation-regulated evaporation recycling services. Formally,

$$\begin{array}{c} \frac{E_{\text{track,current}}-E_{\text{track,desert}}}{E_{\text{current}}}=VMR_{E} \end{array}$$ (3)

where for each grid cell, *E*_track,current_ is the amount of evaporation that is recycled downwind under current vegetation, *E*_track,desert_ is the amount of evaporation that is recycled downwind under desert vegetation, *E*_current_ is the total evaporation from current vegetation, and *VMR*_*E*_ is the ecosystem service associated with evaporation that is both vegetation-regulated and falls as precipitation downwind, as a fraction of total evaporation.

#### Sinks of VMR

Now, we identify the precipitation-recycling ratio by identifying the fraction of precipitation falling in a given grid-cell that originated as evaporation upwind; note that we are using the same approach used in previous studies [7, 8]. The precipitation-recycling ratio is defined for each grid cell as,

$$\begin{array}{c} \frac{P_{\text{c,track}}}{P_{\text{c}}}=\rho_{\text{c}} \end{array}$$ (4)

where, *P*_c,track_ is the precipitation that returns to land as precipitation downwind, *P*_c_ is the total precipitation, and *ρ*_c_ is the terrestrial precipitation recycling ratio.

Next, we identify the extent to which vegetation regulates downwind precipitation by subtracting the tracked precipitation from the desert vegetation scenario from the tracked precipitation from the current vegetation scenario, and dividing this by the total precipitation associated with current vegetation. This generates a ratio we call vegetation-regulated precipitation, where for each grid cell,

$$\begin{array}{c} \frac{P_{\text{track,current}}-P_{\text{track,desert}}}{P_{\text{current}}}=VMR_{P} \end{array}$$ (5)

where, *P*_track,current_ is the amount of precipitation that is generated as upwind land evaporation under current vegetation, *P*_track,desert_ is the amount of precipitation that is generated as upwind land evaporation under desert vegetation, *P*_current_ is the total precipitation, and *VMR*_*P*_ is the ecosystem service associated with precipitation that is both vegetation-regulated and originates as evaporation on land upwind, as a fraction of total precipitation.

## Results

### Global Sources of Vegetation-regulated Moisture Recycling

**Fig 1:**
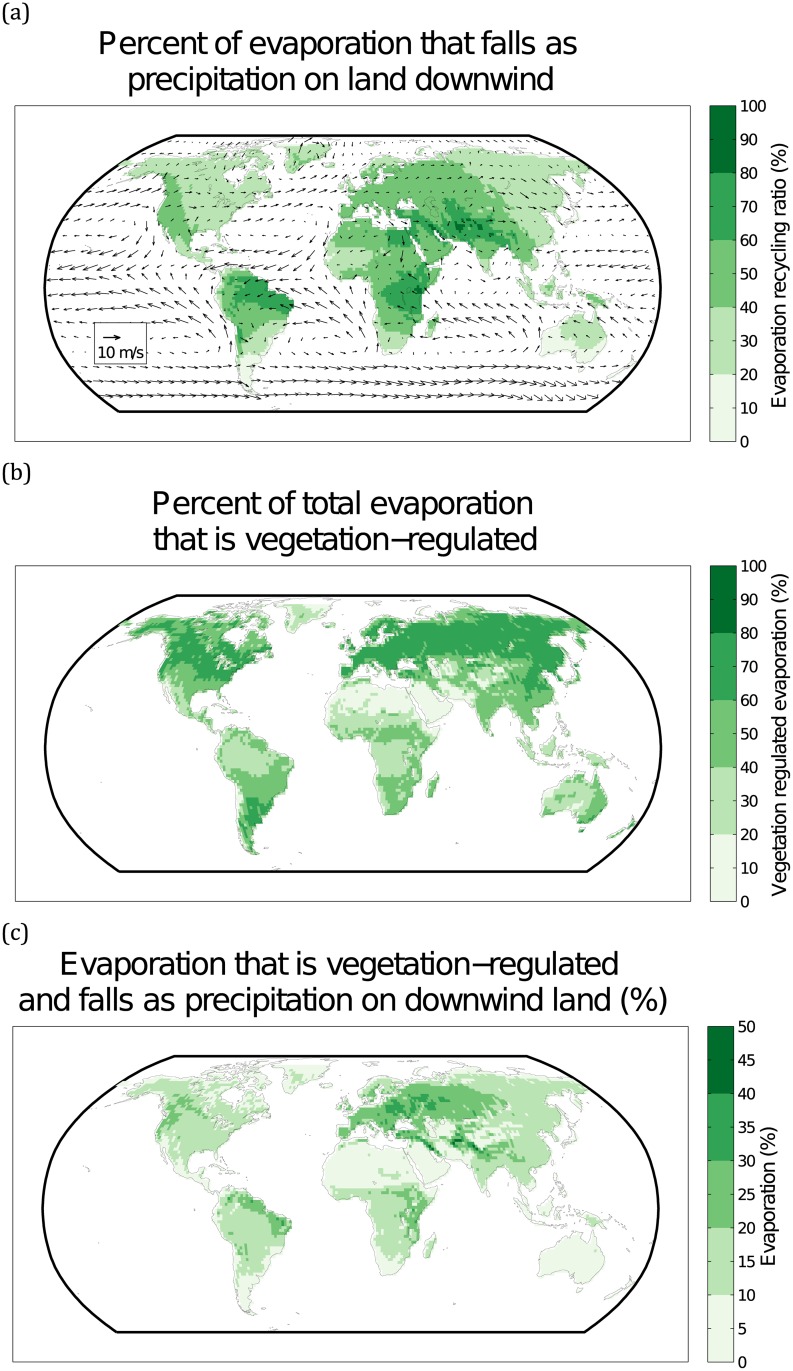
Global Sources of Vegetation-regulated Moisture Recycling, showing the amount of evaporation from land that falls as precipitation on land (as opposed to oceans) downwind (a); the percent of total evaporation that is vegetation-regulated, relative to barren land (b); and the amount of vegetation-regulated evaporation that falls as precipitation on land downwind (c). The arrows in panel (a) depict annual average wind directions in the lower level of the atmosphere. Note that the maximum of (c) is 50%.

The sources of Vegetation-regulated Moisture Recycling (VMR) ecosystem services are identified using both moisture recycling data and vegetation-regulation data. The evaporation recycling ratio is the amount of evaporated water that falls on land versus over the oceans, i.e. *ε*_c_ (Fig 1a). High recycling ratios are particularly evident on the west coast of North America, northeastern Brazil, east Africa, and western Eurasia (ranging from 40 to 80%). There are also some locations in the Tigris-Euphrates valley in Iraq and Syria, and along the mountainous gradient of the Karakorum and Himalaya in south Asia that have particularly high evaporation recycling ratios (80–100%). The average annual, lower-level winds are overlain atop the recycling ratios, and depict the direction and strength of winds as they move around the planet. It is clear that the windward areas of continents (i.e. where winds move from ocean to land), experience the highest rates of evaporation recycling. In other words, due to the prevailing wind direction from west to east in the northern hemisphere, the eastern sides of the large landmasses of North America and Eurasia experience low terrestrial evaporation recycling, since most of the evaporation from these eastern regions returns as precipitation over oceans (rather than land).

The vegetation-regulated evaporation, *V*_*E*_, i.e. the relative difference between evaporation from current versus desert vegetation, appears to be relatively more important in higher latitude regions relative to the tropics (Fig 1b). The lower values of vegetation-regulation found in the tropics may be partly explained by the presence of the Inter Tropical Convergence Zone (ITCZ) as well as monsoonal systems, which bring large amounts of precipitation to land areas, regardless of vegetation. Thus, even without vegetation in the tropics, the oceans and global circulation may still provide moisture to the interiors of tropical continental regions and sustain moisture recycling. Vegetation-regulated evaporation is evidently an important characteristic of much of the Earth’s land surface. More than 85% of the Earth’s land surface has 25% or more of its evaporation regulated by current vegetation. Furthermore, 36% of the Earth’s surface has 50% or more of its evaporation regulated by current vegetation.

By combining the previous two sets of information we are able to calculate the ecosystem service associated with the sources of VMR (Fig 1c). The largest global sources of VMR are found in eastern Europe, the northern Amazon, eastern Africa, and along the Himalaya. At the global scale, the average VMR evaporation ratio for all land areas is 10%. Likewise, approximately 8% of the global land surface experiences VMR evaporation rates of greater than 25%.

### Global Sinks of Vegetation-regulated Moisture Recycling

**Fig 2:**
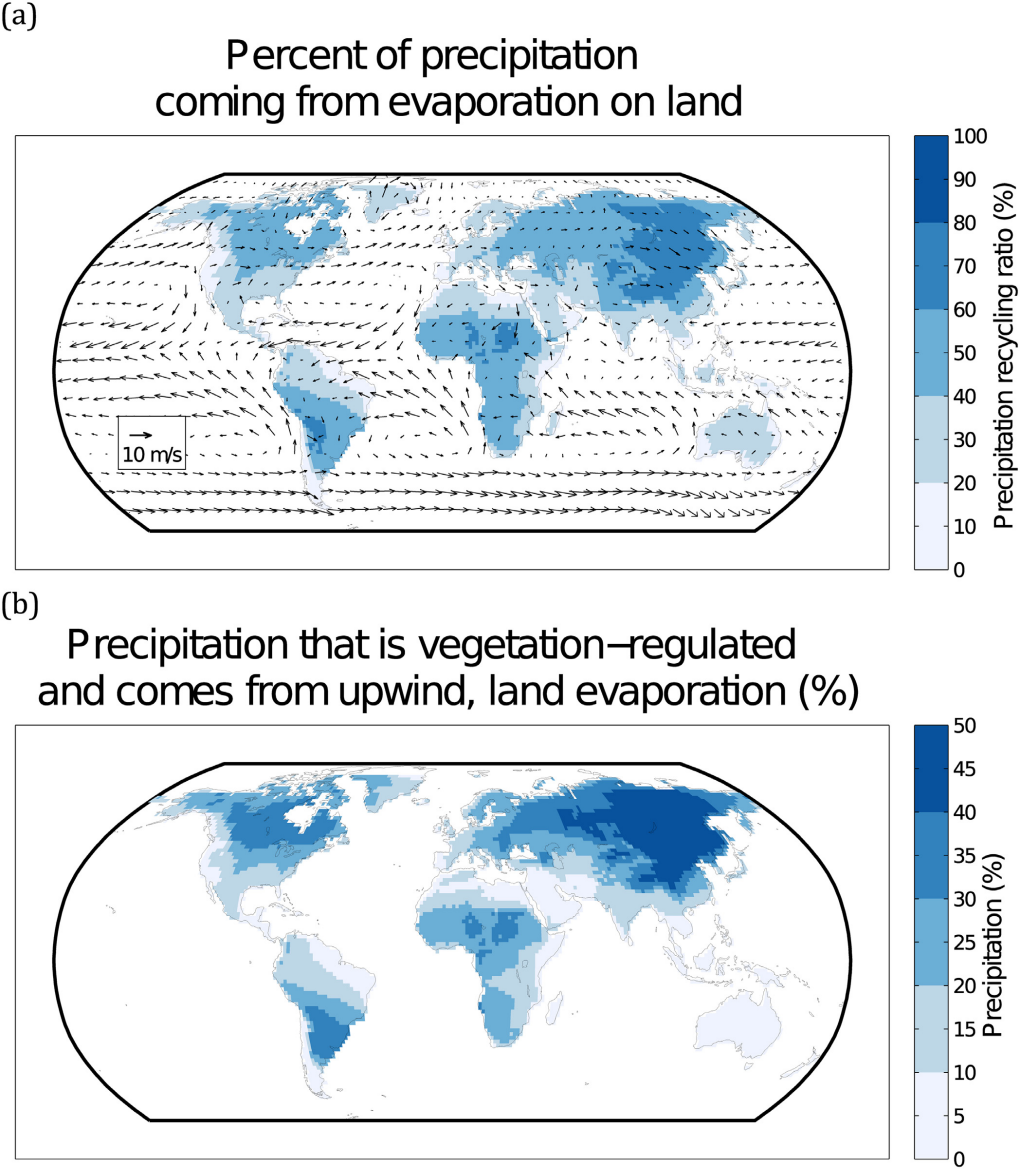
Global Sinks of Vegetation-regulated Moisture Recycling, showing the amount of precipitation on land that originates as upwind evaporation from land (a); and, the amount of precipitation that comes from upwind vegetation-regulated evaporation (b). The arrows in panel (a) depict annual average wind directions in the lower level of the atmosphere. Note that the maximum of (b) is 50%.

The VMR precipitation sinks are the regions that are located downwind from the evaporative sources of VMR (Fig 2). The precipitation recycling ratios (i.e. the ratio of precipitation that comes from land versus oceans; *ρ*_*c*_), are consistent with previous work [9]. The wind directions represent the lower-level atmospheric winds, and indicate that higher precipitation recycling exists on the leeward sides of continents, with East Asia, west and central Africa, and the La Plata region of South America experiencing the highest precipitation recycling ratios. These results are supported by previous work [34], which highlights the fact that oceanic contribution to terrestrial rainfall is very limited for northeastern North America and much of the eastern half of Eurasia.

The global VMR sink regions (Fig 1b), where precipitation is regulated by upwind vegetation, reveal the dependence of East Asian precipitation on VMR ecosystem services. In other words, the evaporation from the mosaic of ecosystems across Eurasia play an important role in sustaining much of the precipitation in northern China, Mongolia, and eastern Siberia. At the global scale, the average VMR precipitation ratio for all land areas is 22%. Additionally, more than 76% of the global land surface receives at least 10% of precipitation from upwind vegetation, a ratio that increases steadily leeward through all populated continents, except Australia. The highest rates are found in central and East Asia, with 40% or more of total precipitation being regulated by upwind vegetation.

### Regional case study and Conceptual Framework

We now know which regions generate VMR and potentially benefit from VMR. A global analysis is critical for understanding how different regions compare with one another, and for comparing with other published work for agreement (or disagreement) in the distribution of VMR generation and benefits. Many ecosystem service analyses are significantly finer in scale and scope, than the entire planet. Thus, we now leave the global scale, and zoom into a more focused case study. This will provide a better sense of how VMR might be used at a more relevant scale and scope compared to other ecosystem service assessments.

**Fig 3:**
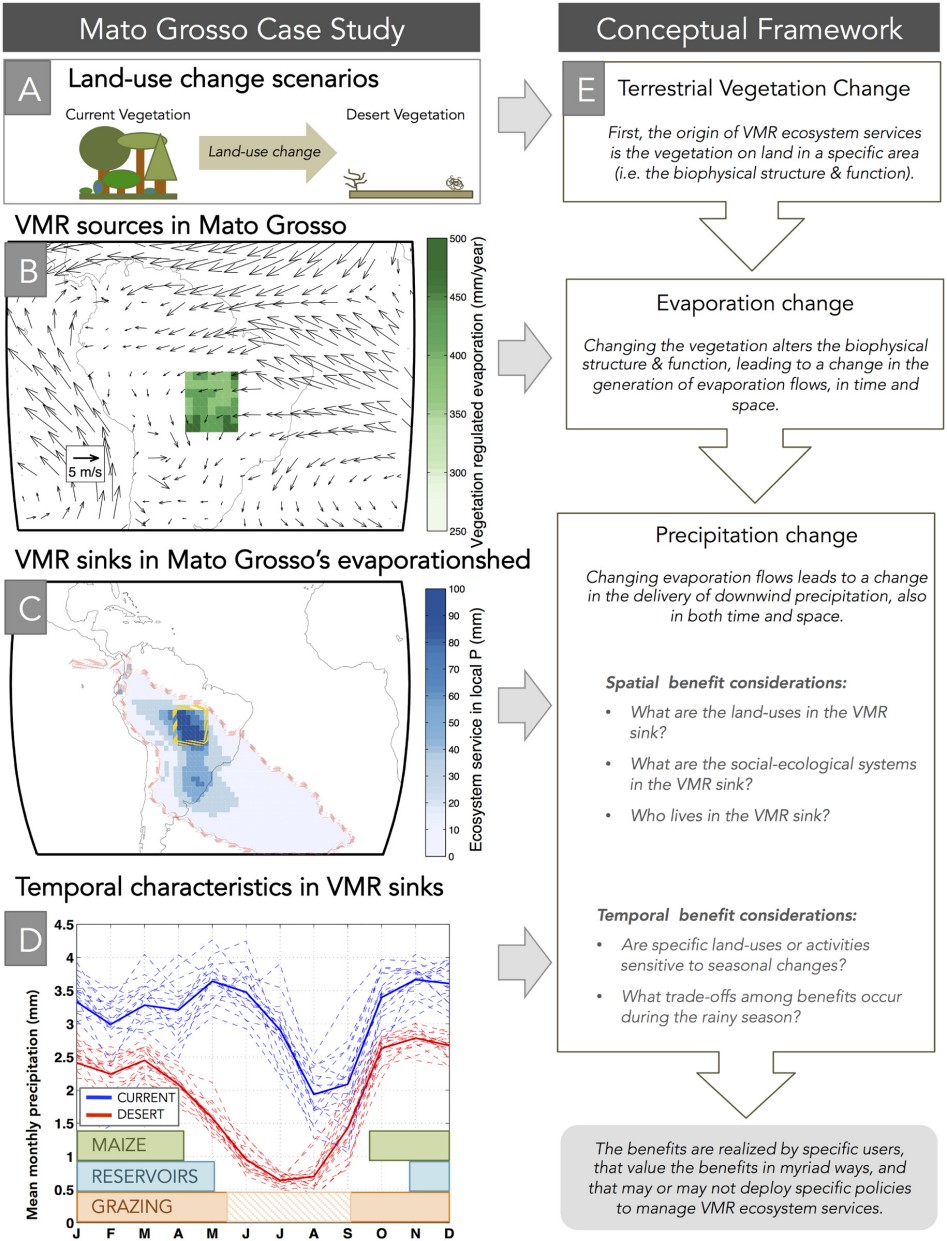
Mato Grosso regional case study and Conceptual Diagram for VMR ecosystem services. A land-use change scenario that replaces current vegetation with desert land (a), is used to derive the amount of vegetation-regulated evaporation (i.e. VMR sources) for the Mato Grosso region of Brazil (b); the arrows in panel (b) depict annual average wind directions in the lower level of the atmosphere. The region downwind of this VMR source is the VMR sink region, also known as the evaporationshed for Mato Grosso (c). The temporal dynamics of VMR precipitation (d), depicting average, monthly variation between the current (blue) and desert (red) vegetation scenarios are shown alongside the seasonal overlap of important sectors affected by precipitation including the growing season for rainfed maize [36], reservoir filling [37], and seasonal rangeland grazing, with the hashed area indicating decreased quality, wintertime forage [38]. This case study forms the basis for our conceptual framework of VMR ecosystem services (e) running parallel to the Mato Grosso figures.

Mato Grosso is a region in Brazil that includes both the Amazonian tropical rainforest, a mosaic of grasslands, croplands, and savannas, and portion of the pantanal. Importantly, Mato Grosso is experiencing rapid land-use change [35], which will alter its evaporation, and consequently the VMR ecosystem services that it provides for regions downwind. Note that we perform an idealized scenario of changing Mato Grosso’s current vegetation to desert vegetation (Fig 3a). This is a similar analysis as before (Fig 2), but at a smaller, regional scale. The Mato Grosso region is here seen as the source of VMR ecosystem services, and the spatial region that benefits from its evaporation is known as the evaporationshed [21]. Essentially, the evaporationshed is the specific downwind area that receives VMR benefits (i.e. precipitation) from upwind VMR generation (i.e. evaporation).

The Mato Grosso analysis reveals several important aspects about the local VMR ecosystem services (Fig 3). First, the vegetation-regulated evaporation, *V*_*E*_, in Mato Grosso is between 300 to 500 mm/yr (calculated as the difference between evaporation from current vegetation and desert vegetation), which amounts to 30 to 45% of total evaporation (Fig 3b). The potential benefits in terms of VMR precipitation (i.e. *VMR*_*P*_) within Mato Grosso itself, range from 60 to 100 mm of annual precipitation, representing up to 6% of total precipitation (Fig 3c). There is a lobe of VMR precipitation that extends directly south, with an annual contribution of 40 to 80mm (representing 2–4% of total precipitation), and a more diffuse area that extends from the central Amazon all the way to the southeastern Brazilian state of Minas Gerais. The rest of the evaporationshed receives between 1 and 20mm of annual precipitation from present-day vegetation in Mato Grosso. Notably, these differences in precipitation between the current and desert vegetation scenarios are well within the range of a recent meta-analysis of 96 regional and global climate model simulations of Amazonian deforestation, which compared how deforestation impacts precipitation [39].

The temporal dimension of this ecosystem service is also important, especially for seasonally-sensitive systems, such as rainfed agriculture, reservoir networks, and livestock grazing. VMR benefits are distinctly different between the current and desert vegetation scenarios, in both seasonal and interannual variability (Fig 3d). Current vegetation (solid blue line) has higher overall VMR, as well as greater interannual variability (dashed blue lines), perhaps indicating how the diverse land-uses respond heterogeneously to variations in climate. Conversely, the desert VMR (solid red line) shows much less interannual variability (dashed red lines), suggesting that the desert vegetation creates a more homogenous response to variations in climate. Finally, the noticeable dip in VMR that occurs in the dry season (i.e. May to September) is wider in the desert scenario, highlighting the importance of vegetation in sustaining moisture recycling into the dry season. This reduction in dry season VMR accounts for approximately 45% of the annual difference between the current and desert vegetation scenarios. We acknowledge that a significant impact to runoff, and subsequently to reservoir storage and management, would also occur with dramatic land-use changes that could lead to additional impacts. We highlight this qualitatively (bars at the bottom of Fig 3d), though we do not explore the consequences to runoff in detail.

From our case study, we develop a conceptual framework that generalizes the important features of how to estimate VMR (Fig 3e, full column). Many ecosystem service frameworks begin with a particular biophysical structure or process, then examine how the service is generated, and eventually identify where and when that service benefits users [40]. In our case, the biophysical structure is the current vegetation, and the ecosystem service is understood as vegetation-regulated moisture recycling, which includes both the areas generating evaporation (i.e. VMR sources) and the areas benefiting from precipitation (i.e. VMR sinks).

## Discussion

We have, for the first time, described Vegetation-regulated Moisture Recycling (VMR) as an ecosystem service. We have also demonstrated its applicability at global and regional scales and generalized these findings to a broadly applicable framework. We now explore the implications of our findings, particularly in the context of broader land-use, hydrological, and governance concerns.

### Overall VMR Patterns

At the global scale, the lower importance of VMR in the tropics controverts much popular wisdom about the role of tropical vegetation for sustaining terrestrial moisture recycling [41, 42]. The temperate regions of North and South America, and temperate Eurasia experience the highest rates of VMR (Figs 1c and 2b). Additionally, it is important to consider that our global analysis (Figs 1 and 2) is based on annual average characteristics, so some interannual variability is masked. This is clearly visible in the Mato Grosso case study, where VMR plays a particularly important role during the dry season (Fig 3d). The pronounced gap between the blue line (current vegetation) and red line (desert vegetation) during the dry season (May to September) indicate the importance of Mato Grosso’s current vegetation for sustaining dry-season rainfall. This dry-season supply of moisture further underscores the importance of evaporation processes unique to vegetation, such as transpiration [18].

### Land-use change affecting VMR sources

The regions with particularly high rates of VMR source evaporation (Fig 1c) emphasize the importance of current vegetation in sustaining downwind precipitation, and that significant land-use change could adversely affect VMR ecosystem services. The intense source areas in Europe are not likely to experience dramatic land-use change, given the maturity of those economies [43], although agricultural land abandonment is an increasingly important phenomena [44]. Large agricultural expansions are similarly unlikely in North America and western Russia, yet both of these regions could potentially experience evaporation reductions given persistent and widespread forest fires that interrupt forest succession, and force ecosystems to transition into lower-evaporation regimes [45, 46]. Likewise, pervasive land degradation (e.g. soil salinization, topsoil erosion) and land-use change in east Africa and south Asia could potentially reduce VMR evaporation [47].

Our case study reveals the importance of Amazonia (particularly Mato Grosso), a region often discussed in relation to moisture recycling [23, 48] for sustaining potential VMR benefits in parts of South America. Though the regional benefits are diffuse, some regions receive between 2 and 6% of total rainfall from this area. Likewise, the temporal dimension (see Fig 3d) highlights not only the overall precipitation delivered by VMR, but also the persistence of moisture delivery into the dry, winter/spring season (May-September). This difference in dry season moisture supply is especially important for fragile systems that are already stretched to the brink, such as the Sistema Cantareira, which brings drinking water to the Sao Paulo metropolitan area [49].

### Water scarcity in VMR beneficiary regions

Regions with high VMR benefits (Fig 2c) coincide with many areas under current, and projected, water scarcity [22, 50]. Much of sub-Saharan Africa receives between 10–30% of precipitation from VMR, with some areas as high as 40% or more. This dependence on upwind VMR is striking given sub-Saharan Africa’s continued reliance on subsistence, rainfed agriculture. As populations grow and available land shrinks, the vulnerability of sub-Saharan Africa to any reduction in VMR ecosystem services increases substantially [51]. At the other end of the rural to urban gradient, many global megacities receive drinking water from regions that rely 40% or more on VMR [52]. To use our case study region as an example, there are three megacities within the evaporationshed (Buenos Aires, Sao Paulo, and Rio de Janeiro), of which the latter two are currently experiencing an unprecedented water crisis. Despite a VMR dependence of between 20 and 30% (for Sao Paulo and Rio de Janeiro, respectively; Fig 2b), even small changes in precipitation arising from upwind land-use change could have big impacts to the fragility of urban water supplies.

### Governance challenges for VMR ecosystem services

The diffuse nature of VMR leads to potential difficulty in relating biophysical service generation in one place with specific, tangible, and traceable benefits downwind. Nonetheless, existing literature related to diffuse ecosystem services, such as pollination, air pollution, and migratory animal species, could provide useful analogs for policy intervention [53, 54]. In particular, spatial information can facilitate inter-comparison among overlapping ecosystem services, and the trade-offs that arise from pursing different management strategies [55]. Our analysis provides both spatial and temporal information that can be used to compare VMR benefits with other ecosystem services with overlapping spatial or temporal attributes. The governance of terrestrial moisture recycling services is also likely to be challenging, due to the diffuse and spatially extensive nature of the service. Yet, there are multiple existing methods of governance for transboundary phenomena such as trade pacts, resource treaties, and collective ecological priorities [56, 57].

## Conclusions

Vegetation-regulated Moisture Recycling (VMR) is an ecosystem service. We have demonstrated a method for quantifying this at global and regional scales, where vegetation regulates the atmospheric branch of the water cycle, specifically evaporation and precipitation. We have also provided a broadly applicable framework for incorporating VMR into existing ecosystem services assessments. This is very important given that the use of ecosystem services has become a cornerstone of natural resource management and sustainability efforts. Framing the regulation of atmospheric moisture as an ecosystem service that is both influenced by humans and has the potential to impact downwind human societies, can improve future analyses of ecosystem services related to hydrological change, large-scale land-use change, and land-atmosphere tele-connections.
